# A Multiplex RT-QPCR Assay of ONECUT2, EZH2, and BRCA2 for the Detection of Clinically Significant Prostate Cancer in Needle Biopsies

**DOI:** 10.3390/cancers18182962

**Published:** 2026-09-14

**Authors:** Yuta Inoue, Steven Yong Cen, Masatomo Kaneko, Gangjun Yuan, Zhenzhong Deng, Rongying Lu, Michelle C. Gong, Jerry Zhang, Federico Eskenazi, Shiran Konganige, Manju Aron, Yohei Sekino, Tsuyoshi Iwata, Atsuko Fujihara, Osamu Ukimura, Inderbir Gill, Andre Luis Abreu, Gangning Liang

**Affiliations:** 1 Department of Urology, USC Norris Comprehensive Cancer Center, University of Southern California, Los Angeles, CA 90089, USA; 2 Department of Urology, Kyoto Prefectural University of Medicine, Kamigyo-Ku, Kyoto 602-8566, Japan; 3 Department of Radiology/Neurology, University of Southern California, Los Angeles, CA 90089, USA; 4 Department of Urology, Chongqing University Cancer Hospital, Chongqing 400044, China; 5 Department of Pathology, USC Norris Comprehensive Cancer Center, University of Southern California, Los Angeles, CA 90089, USA; 6 Department of Urology, Graduate School of Biomedical Sciences, Hiroshima University, Hiroshima 739-0046, Japan

**Keywords:** prostate cancer, needle biopsy, multiplex RT-qPCR, DNA methylation, ONECUT2, EZH2, BRCA2

## Abstract

**Background/Objectives**: Needle biopsy-based molecular assays that are sensitive, cost-effective, and reproducible could improve prostate cancer (PCa) diagnosis and risk stratification. We developed a compact, biologically informed gene expression signature panel (ONECUT2, EZH2, BRCA2) for cancer detection and aggressiveness stratification. **Methods**: We designed and validated a multiplex real-time RT-qPCR assay for ONECUT2, EZH2, and BRCA2. Biomarker selection was guided by The Cancer Genome Atlas Prostate Adenocarcinoma (TCGA-PRAD) and Gene Expression Omnibus (GEO) cohort analyses. The assay was applied to 979 ex vivo needle biopsy cores from 255 radical prostatectomy specimens; machine-learning models (Random Forest, AdaBoost, Elastic Net) generated an RNA signature. Diagnostic and prognostic performance was assessed by the area under the receiver operating characteristic curve (AUC), model comparisons by DeLong’s test, and biochemical recurrence-free survival (BCR) by Kaplan–Meier and Cox proportional hazards analyses. Reproducibility based on sample pairs was assessed by intraclass correlation coefficients (ICC). **Results**: The three-gene expression signature (ONECUT2, EZH2, and BRCA2) robustly discriminated malignant from non-malignant tissue (AUC = 0.91) and identified clinically significant cancer (Gleason Grade Group [GG] ≥ 2 vs. GG 0–1, AUC = 0.89) within biopsy cores. However, it had limited performance for advanced patient-level features (e.g., pathological GG3–5, AUC = 0.63). In contrast, ONECUT2 gene-body methylation significantly stratified pathological aggressiveness (pathological GG3–5, AUC = 0.79; aggressive disease, AUC = 0.75) and demonstrated superior assay stability (methylation ICC 0.58–0.70 vs. RNA ICC 0.38–0.61). Furthermore, ONECUT2 DNA methylation significantly stratified BCR-free survival (hazard ratio = 3.90, *p* = 0.023), whereas the RNA signature showed a modest trend (hazard ratio = 1.88, *p* = 0.053). Notably, integrating DNA methylation with the three-gene expression signature (combined model) significantly improved predictive accuracy for adverse features (e.g., extraprostatic extension, ΔAUC = 0.20, *p* = 0.02) compared to the three-gene expression signature alone. **Conclusions**: While a multiplex RT-qPCR panel (ONECUT2, EZH2, and BRCA2) excels at core-level cancer detection, it lacks the resolution for aggressiveness classification. In contrast, ONECUT2 DNA methylation provides superior analytical reproducibility, robust stratification of cancer aggressiveness, and long-term recurrence risk estimation. These findings suggest that integrating transcriptional and epigenetic markers into a multimodal framework could provide complementary information to refine risk stratification in PCa.

## Simple Summary

A prostate cancer diagnosis often relies on needle biopsies, but these methods sometimes fail to distinguish between indolent and highly aggressive tumors accurately, which can lead to overtreatment or delayed intervention. In this study, we developed a fast, cost-effective genetic test, a multiplex RT-qPCR assay, that detects the presence of clinically significant cancer by measuring three specific genes in small biopsy samples. While this gene test was somewhat insufficient for evaluating tumor aggressiveness, focusing on a DNA modification marker called DNA methylation revealed that DNA methylation is superior for predicting disease aggressiveness and long-term recurrence. Combining both markers creates a powerful tool that helps clinicians more reliably refine treatment decisions, such as identifying candidates for active surveillance or guiding timely intervention. This approach could easily be used in standard clinical laboratories to improve personalized treatment decisions for prostate cancer patients.

## 1. Background

Prostate cancer (PCa) is the most frequently diagnosed malignancy and the second leading cause of cancer-related death among men worldwide [1]. The clinical behavior of PCa is highly heterogeneous, ranging from indolent tumors to aggressive, lethal phenotypes [2]. While traditional clinicopathological parameters, such as prostate-specific antigen (PSA) and Gleason Grade Group (GG) [3], serve as the cornerstone of risk stratification, they often fail to capture the underlying molecular complexity of the tumor, leading to potential overtreatment of low-risk disease or undertreatment of aggressive variants [4].

PCa exhibits marked genetic and epigenetic heterogeneity, making both diagnosis and treatment particularly challenging. Genetic and epigenetic alterations—including genetic mutations, copy number variations, DNA methylation, chromatin remodeling, and RNA dysregulation—play key roles in tumor initiation, progression, and therapeutic resistance, while also providing valuable opportunities for biomarker development. Liquid biopsy approaches, including circulating tumor DNA, circulating tumor cells, and exosomes, enable minimally invasive and real-time assessment of tumor dynamics and resistance pathways, complementing conventional biomarkers like PSA and advancing precision oncology strategies. The incorporation of epigenetic and liquid biopsy biomarkers into multimodal diagnostic frameworks holds significant promise for personalized PCa management [5,6]. However, most current diagnostic assays, including liquid biopsies, lack spatial information about tumor localization. In contrast, prostate needle biopsy provides essential tumor-specific data, including molecular characteristics and precise anatomical context. Therefore, despite advances in imaging, histopathological confirmation through prostate core needle biopsy remains indispensable in clinical practice. Although several studies, including those from our group, have explored gene expression and DNA methylation biomarkers for the diagnosis and prognosis of PCa [7,8,9,10,11,12,13,14,15,16,17], practical biomarkers specifically designed for needle biopsies remain underdeveloped. Consequently, there is a critical need for methodologies that are highly sensitive, cost-effective, and reproducible.

To address this challenge, following an initial in silico screening of 16 candidate prognostic genes using public datasets (TCGA-PRAD), we developed a simplified multiplex real-time RT-qPCR system targeting three biologically distinct but clinically synergistic drivers of aggressive PCa: ONECUT2, EZH2 and BRCA2. The rationale for selecting this “compact panel” from the initial candidates lies in their ability to represent not only the key hallmarks of aggressive disease progression but also therapeutic vulnerability.

First, ONECUT2 is a master transcriptional regulator that suppresses the androgen receptor program and drives neuroendocrine differentiation, a hallmark of lethal, treatment-resistant disease [18,19]. Second, EZH2, a subunit of the Polycomb Repressive Complex 2, promotes epigenetic reprogramming and epithelial–mesenchymal transition, serving as a robust marker of metastasis and recurrence [20,21]. Third, BRCA2 is crucial for homologous recombination DNA repair; its deficiency not only predicts poor survival and aggressive histology but also serves as a critical predictive biomarker for response to poly (ADP-ribose) polymerase (PARP) inhibitors and platinum-based chemotherapy [22,23].

In the current study, we describe the development of this multiplex RT-qPCR assay and evaluate its feasibility and diagnostic performance using ex vivo prostate needle biopsy samples from radical prostatectomy specimens. The primary clinical objective of this assay is to provide a sensitive and cost-effective molecular tool for core-level cancer detection and the identification of clinically significant cancer, which could complement routine pathology in standard clinical laboratories. Furthermore, as a secondary objective to evaluate its broader prognostic utility, we investigated its ability to predict patient-level advanced pathological features. Building on our recent study demonstrating the prognostic potential of ONECUT2 gene body methylation [15], we also performed a comparative analysis between ONECUT2 DNA methylation status and the multiplex expression of ONECUT2, BRCA2, and EZH2. Taken together, although the multiplex RT-qPCR panel (ONECUT2, EZH2, and BRCA2) demonstrates high sensitivity for biopsy-level cancer detection (our primary objective), the combination of DNA methylation with the three-gene expression signature significantly improved predictive accuracy for these advanced patient-level features compared to the three-gene expression signature alone.

## 2. Methods

### 2.1. Bioinformatics Analysis and Validation in Public Datasets

For The Cancer Genome Atlas Prostate Adenocarcinoma (TCGA-PRAD) cohort analysis, we performed a comprehensive in silico analysis to validate the prognostic significance and clinical relevance of the selected genes [24].

Gene expression data (RNA-Seq, STAR-Counts) and corresponding clinical information for PCa were obtained from the GDC Data Portal using the TCGAbiolinks package (version 2.38.0) in R. Raw count data were normalized to Transcripts Per Million (TPM) and subsequently log2-transformed (log_2_(TPM + 1)) to stabilize variance. Samples were classified into malignant and non-malignant tissues based on the TCGA sample type codes. For clinicopathological analyses, pT stages were consolidated into T2, T3 and T4, and GG were categorized into groups ranging from 1 to 5 [3]. For survival analysis, tumor samples were first stratified into high- and low-expression groups based on the median expression value of each specific gene (ONECUT2, EZH2 and BRCA2). Next, the total number of highly expressed genes (ranging from 0 to 3) was determined for each patient.

### 2.2. Validation in Gene Expression Omnibus (GEO) Datasets

To validate the clinical relevance of ONECUT2, EZH2 and BRCA2 expression, we analyzed five independent transcriptomic datasets retrieved from the NCBI Gene Expression Omnibus (GEO) database: GSE3325 [25], GSE6919 [26,27], GSE21034 [28], GSE35988 [29] and GSE32269 [30].

Raw expression data were downloaded using the GEOquery package (version 2.78.0). Probe IDs were mapped to gene symbols based on the platform annotation. To ensure robust quantification, when multiple probes mapped to a single gene, the median expression value of those probes was calculated and used as the representative expression level for that gene. Samples were stratified into clinical or histological subgroups (e.g., Benign/Normal, Localized PCa, and Metastatic PCa/castration-resistant PCa (CRPC)) based on the metadata provided in each dataset.

### 2.3. Patients and Ex Vivo Sampling

**Figure 1:**
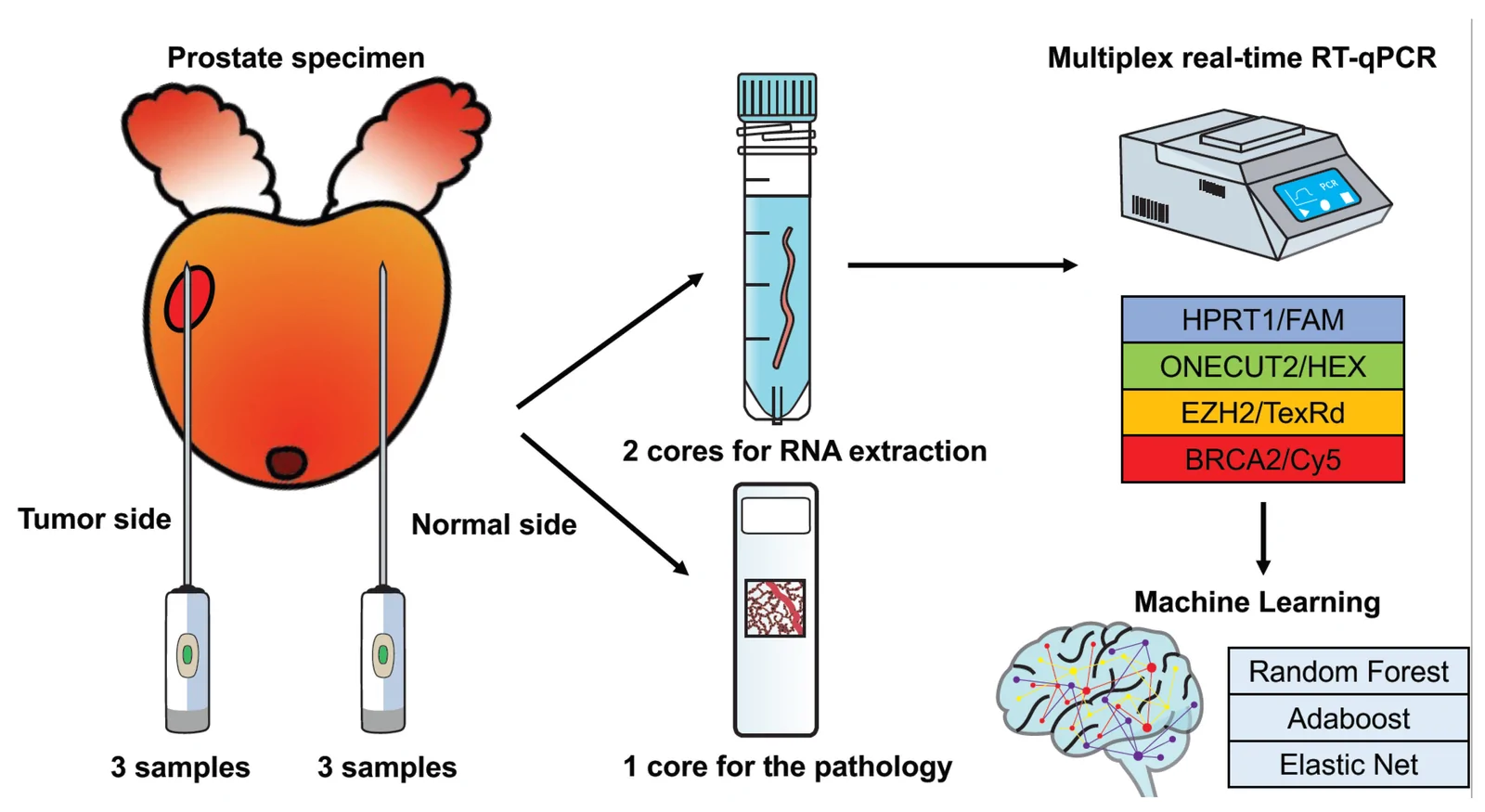
Schematic overview of the ex vivo prostate needle biopsy sampling and molecular analysis workflow. Ex vivo needle biopsies are performed on fresh radical prostatectomy specimens. As illustrated for prostate cancer cases, three biopsy cores are collected from the index tumor lesion (tumor side), and three cores are sampled from the contralateral non-malignant side (normal side). From each sampling site, one core is processed for standard histopathological evaluation (H&E staining) to confirm the tissue status and assign an ex vivo Gleason Grade Group. The remaining two cores are immediately preserved for RNA extraction. The extracted RNA is subsequently analyzed using a custom multiplex real-time RT-qPCR assay targeting ONECUT2, EZH2, and BRCA2, alongside the endogenous reference gene HPRT1, utilizing distinct fluorophores: FAM (blue), HEX (green), Texas Red (yellow), and Cy5 (red). Finally, the normalized gene expression data are utilized to train and evaluate machine learning models (Random Forest, AdaBoost, and Elastic Net) to predict cancer presence and tumor aggressiveness.

A schematic overview of the ex vivo prostate needle biopsy sampling and molecular analysis workflow is described in Figure 1. Between April 2019 and February 2025, patients with PCa undergoing radical prostatectomy (with/without bilateral pelvic lymph node dissection) and those with symptomatic benign prostatic hyperplasia (BPH) undergoing simple prostatectomy were prospectively recruited for this study under an Institutional Review Board-approved protocol (IRB# HS-16-00404). In accordance with standard-of-care guidelines and at the treating urologist’s discretion, bilateral pelvic lymph node dissection was performed in patients with intermediate- or high-risk disease [31]. Ex vivo needle biopsies were obtained from fresh surgical specimens immediately following resection. Sampling was guided by cognitive fusion based on preoperative MRI and biopsy pathology to target the index lesion. Specifically, for PCa cases, three cores were collected from the index tumor lesion, and three cores were sampled from the contralateral non-neoplastic region (control). For patients with BPH serving as pure non-malignant controls, three cores were systematically sampled from a single lobe, as no index lesion was present.

From each sampling site, one core was processed for histopathological analysis (H&E staining) by a pathologist to confirm tissue status and to assign an ex vivo GG to each core. The remaining two cores were immediately preserved in DNA/RNA Shield (Zymo Research, Irvine, CA, USA) to stabilize nucleic acids, de-identified, and subsequently processed for RNA and DNA extraction. The radical prostatectomy specimens were processed according to the standard of care and ISUP guidelines [3]. Initially, a total of 1019 biopsy cores were collected from a cohort of 270 patients. Following rigorous quality control of the extracted nucleic acids, 979 cores from 255 patients yielded high-quality RNA and were ultimately included in the gene expression analysis. This final cohort included 17 cores from 9 patients with benign prostatic hyperplasia, which served as non-malignant controls. Furthermore, a specific subset of this RNA-analyzed cohort (261 samples from 80 patients) was utilized for the DNA methylation analysis. Comprehensive clinical and pathological data were collected for each patient, including age, race, ethnicity, preoperative PSA, PSA density, prostate volume, clinical T stage, routine clinical biopsy GG, National Comprehensive Cancer Network (NCCN) risk group, and Cancer of the Prostate Risk Assessment (CAPRA) score [31,32]. Furthermore, final pathological features from the RP specimens were recorded, including pathological GG (pGG), pathological T (pT) stage, pathological N (pN) stage, extraprostatic extension, seminal vesicle invasion, intraductal carcinoma, cribriform architecture, bladder neck invasion, lymphovascular invasion, and perineural invasion according to ISUP [3]. Patients who underwent RP were monitored with PSA testing every 6 to 12 months for 5 years and then annually. Imaging studies were performed to rule out local or distant recurrence when biochemical recurrence (BCR), defined as PSA persistence or two consecutive PSA values greater than 0.2 ng/mL, was observed.

### 2.4. Cell Lines and Cell Culture

RWPE1 (a non-tumorigenic parental prostate line originating from non-malignant human prostate epithelium), C4-2B, PC3 and LNCaP (PCa cell lines) were obtained from the American Type Culture Collection (ATCC, Manassas, VA, USA; www.atcc.org (accessed on 3 December 2024)). RWPE1 (CRL-3607™) cells were cultured in keratinocyte serum-free medium containing 50 μg/mL bovine pituitary extract and 5 ng/mL epidermal growth factor (Thermo Fisher Scientific, Waltham, MA, USA). C4-2B (CRL-3315™), LNCaP (CRL-1740™) and PC3 (CRL-1435™) cells were cultured in RPMI1640 (Corning Inc., Corning, NY, USA), 10% Fetal Bovine Serum (Corning) and 1% penicillin-streptomycin (Genesee Scientific, El Cajon, CA, USA).

### 2.5. RNA Extraction and Precipitation

Total RNA from RWPE1, LNCaP, PC3 and C4-2B cells was extracted using the Qiagen RNeasy Mini Kit (Qiagen, Hilden, Germany; Cat.:74106) according to the manufacturer’s instructions. Total RNA from the ex vivo biopsy specimen was extracted from the patient cores using the Quick-DNA/RNA™ Miniprep Plus Kit (Zymo Research) according to the manufacturer’s instructions. All extracted RNA samples were immediately aliquoted and stored at −80 °C until further downstream processing. Total RNA samples were precipitated through the addition of 0.1 volumes of 3M sodium acetate (pH 5.2) (Corning), 2.5 volumes of 100% ethanol, and 40 µg of glycogen (Avantor, Radnor, PA, USA) as a carrier. Following a 30 min incubation at −80 °C, the RNA was pelleted by centrifugation at 15,000× *g* for 20 min at 4 °C. The pellet was subjected to a thorough cleansing process involving a 70% ethanol wash, followed by an air-drying procedure. Thereafter, the pellet was resuspended in RNase-free water. RNA concentration was measured with a NanoPhotometer^TM^ Pearl (Implen, Munich, Germany). A median of 1345 ng of total RNA (interquartile range [IQR]: 560–2574) was obtained.

### 2.6. Primers and Probes Construction

Specific primers and hydrolysis probes for the three target genes (ONECUT2, EZH2, BRCA2) and the reference gene (HPRT1) were designed using Primer-BLAST (NCBI, Bethesda, MD, USA; https://www.ncbi.nlm.nih.gov/tools/primer-blast/ (accessed on 15 February 2025)) and the PrimerQuest Tool (Integrated DNA Technologies, Coralville, IA, USA; https://www.idtdna.com/PrimerQuest/Home/Index (accessed on 17 February 2025)). To prevent the amplification of genomic DNA, all primer pairs were specifically configured to span exon–exon junctions (Table S1).

Given the multiplex nature of the assay, the compatibility of all oligonucleotides was strictly evaluated using the Multiple Primer Analyzer (Thermo Fisher Scientific; https://www.thermofisher.com/us/en/home/brands/thermo-scientific/molecular-biology/molecular-biology-learning-center/molecular-biology-resource-library/thermo-scientific-web-tools/multiple-primer-analyzer.html (accessed on 23 February 2025)).

### 2.7. Reverse Transcription and Multiplex Real-Time qPCR

Total RNA (up to 1 μg) was reverse-transcribed into cDNA using SuperScript III Reverse Transcriptase (Invitrogen, Carlsbad, CA, USA). For the multiplex qPCR assay, fluorescent probes were labeled with distinct fluorophores for simultaneous detection: FAM (HPRT1), HEX (ONECUT2), Texas Red (EZH2), and Cy5 (BRCA2) (Integrated DNA Technologies). Multiplex qPCR was performed using PrimeTime™ Gene Expression Master Mix (Integrated DNA Technologies) on a CFX96 Touch Real-Time PCR Detection System and a CFX Opus 96 Real-Time PCR instrument (Bio-Rad Laboratories, Hercules, CA, USA). The thermal cycling conditions consisted of an initial denaturation step at 95 °C for 3 min, followed by 40 cycles of 95 °C for 15 s and 60 °C for 30 s.

All samples were run in duplicate. A standard curve was generated using serially diluted cDNA derived from the PC3 cell line to evaluate PCR efficiency and linearity. cDNA from the LNCaP cell line was included in every plate solely as an inter-run calibrator and was not used for direct comparison with the clinical samples. Furthermore, cDNA from C4-2B and RWPE1 cell lines was included in every run as positive and negative controls, respectively. Finally, no-template and minus-reverse transcriptase (no-RT) controls were run in parallel to monitor for contamination and genomic DNA carryover; as expected, neither exhibited amplification.

### 2.8. RT-qPCR Data Processing and Quality Control

Raw amplification data were processed using a custom bioinformatics pipeline. To exclude technical artifacts, an automated quality control algorithm was applied to the relative fluorescence unit (RFU) of each well. Baseline stability was checked for irregular drifts or high noise in the baseline cycles (cycles 3–10). Signal amplitude was evaluated to ensure that the fluorescence increase exceeded a minimum signal-to-noise threshold (defined as >5 standard deviations of the baseline). Furthermore, exponential growth was verified by confirming a sustained positive slope and the absence of abnormal post-peak decay (hook effect or signal drop). Wells failing any of these criteria were flagged and excluded from downstream analysis.

Additionally, samples with a quantification cycle (Cq) value > 35 for the reference gene (HPRT1) were considered low-quality and excluded. Standard curves were constructed by plotting Cq values against the log10-transformed concentrations of the PC3 dilution series. The PCR efficiency (*E*) for each target was calculated using the formula: *E* = (10^−1/slope^ − 1) × 100. Assays showing an efficiency between 85 and 115% and a linearity (*R*^2^) ≥ 0.98 were considered acceptable for quantification.

Relative gene expression was determined using the standard curve method. First, the quantity of each target gene was calculated based on its specific standard curve. These values were then normalized to the quantity of the endogenous reference gene (HPRT1). Finally, the normalized expression levels were calibrated relative to the LNCaP sample (set to 1) to allow for cross-comparison between samples.

### 2.9. Study Endpoints

To define the intended clinical use of our assay, we evaluated the diagnostic and prognostic performance of the biomarker signatures by stratifying the outcomes into primary and secondary clinical endpoints:

Primary Endpoints (core-level detection):

(i) the presence of PCa (ex vivo GG ≥ 1) in ex vivo biopsy cores; and (ii) the presence of clinically significant PCa (ex vivo GG ≥ 2) in ex vivo biopsy cores. Secondary Endpoints (patient-level prognostic evaluation):

(iii) pGG ≥ 3; (iv) pT ≥ 3; (v) lymph node metastasis (pN1); (vi) a composite aggressive feature (defined as pGG ≥ 3, pT ≥ 3, or pN1) [33]; and (vii) BCR-free survival after RP (evaluated as a time-to-event endpoint).

## 3. Statistical Method

### 3.1. Public Data Validation

TCGA-PRAD and GEO data processing and statistical analyses were performed using R software (version 4.5.2). Differential gene expression between malignant and non-malignant tissues was evaluated using the Wilcoxon rank-sum test. The associations between gene expression levels and clinicopathological parameters, including pT stage and GS, were assessed using the Kruskal–Wallis H test. For comparisons involving three or more groups (GSE3325, GSE6919, GSE21034, GSE35988), statistical significance was evaluated using the Kruskal–Wallis test, followed by a post hoc test with Benjamini–Hochberg (BH) correction for multiple comparisons. For comparisons between two groups (Localized vs. CRPC in GSE32269), the Wilcoxon rank-sum test was utilized. Survival probability was estimated using the Kaplan–Meier method stratified by the total number of highly expressed genes (ranging from 0 to 3) and compared using the log-rank test (TCGA-PRAD). All visualizations were generated using the ggplot2 (version 4.0.3) and ggpubr (version 0.6.3) packages. *p*-values adjusted for multiple comparisons (where applicable) were considered statistically significant. A *p*-value of <0.05 was considered statistically significant.

### 3.2. Assay Reproducibility

To assess the reliability and reproducibility of the biomarker measurements (both the three-gene expression signature and ONECUT2 DNA methylation), we analyzed duplicate ex vivo biopsy cores obtained from the same patients. The consistency between the duplicate samples was evaluated by calculating the intraclass correlation coefficient (ICC) using a two-way mixed model with absolute agreement by single measures (ICC 3,1) [34]. An ICC value closer to 1 indicates higher reproducibility and assay reliability. Additionally, to directly compare the assay stability between modalities, the absolute measurement errors (differences between replicates) for each sample were calculated and compared using a paired Wilcoxon signed-rank test.

### 3.3. Machine Learning Algorithms

Three machine learning (ML) algorithms were used to build classifiers: Random Forest (RF), Real AdaBoost [35], and Elastic Net. RF and AdaBoost are considered non-parametric approaches while Elastic Net is considered a parametric approach in cases of strong linear predictors. Each of the three machine learning algorithms was trained and cross-validated independently for each outcome. The hyperparameter setting for RF was based on grid search using an interval of 5, 10, 25, 50, and 100 variables to enter and 200 to 1000 trees by an interval of 100. Other hyperparameters were set as follows: a maximal depth of 50 and a leaf size of 5. The optimized hyperparameter was selected by minimizing the out-of-bag misclassification rate. For Adaboost, since it is more efficient, only 25 trees were built with a depth of 3, as recommended by Hastie et al. [36]. For Elastic Net, the α value was optimized by gradient descent in the range of 0.1 to 0.02 by 0.001, and the hyperparameter tuning for L1 ratio was performed between 0 and 1 by increments of 0.1 to minimize cross-validation predicted residual sum of squares (CVPRESS). For Random Forest and Adaboost, Gini impurity index was used as the loss function. The Loh method with intensive chi-square computing was used for variable selection [37]. Cross-validation predicted residual sum of squares (CVPRESS) was used for Elastic Net to select candidate predictors as well as the final model. For imbalanced outcomes, prior correction as described by King et al. was used [38]. For all 3 classifiers, 10-fold cross-validation was used to evaluate model performance and prevent optimistic bias. The full dataset was equally divided into 10 folds. Within each iteration, 90% of the data were used for model fitting, hyperparameter tuning, and variable selection, while the remaining 10% served as an independent held-out test fold. We reiterated the learning process 10 times and applied the classifier to each of the testing samples. Thus, each study sample served as an independent testing case once (out-of-fold testing). Machine learning models were trained independently for each core-level and patient-level endpoint. For patient-level pathological endpoints (such as pathological GG, pT stage, pN status, composite aggressive disease, and BCR), multiple biopsy cores obtained from the same patient were evaluated, and the maximum predicted probability (maximum risk score) among the cores from that patient was used as the representative patient-level prediction. This maximum-value aggregation strategy aligns with standard clinical pathology principles (where the highest Gleason grade determines the overall patient grade) and captures the highest biological risk within heterogeneous tumor tissues. Prediction accuracy was quantified using the area under the receiver operating characteristic curve (AUC) with 95% confidence intervals (CIs). To report the optimal and most robust performance, the presented ROC curves and AUC values reflect the best-performing model selected among the three algorithms for each specific outcome based on cross-validation performance. We determined the optimal threshold that maximized the AUC for each model with 95% Clopper-Pearson CIs. To evaluate whether combining RNA expression and DNA methylation provided independent, incremental predictive value beyond single-modality baseline models, we tested the differences between full (Combined) and reduced models (ΔAUC) using DeLong’s test for paired ROC curves. Statistically significant improvements in AUC (ΔAUC, *p* < 0.05) indicate that the added molecular biomarker contributes independent predictive value to the classification. For the time-to-event endpoint (BCR-free survival), survival probabilities were estimated using the Kaplan–Meier method and compared using the Log-rank test. Univariate Cox proportional hazards regression was performed to calculate hazard ratios (HR) with 95% confidence intervals (CIs), and predictive discrimination was evaluated using the concordance index (C-index). SAS Enterprise Miner 15.1: High-Performance procedures were used for machine learning. Time-to-event endpoint analyses were performed using R software (version 4.5.2).

## 4. Results

### 4.1. Selection of Target Genes Based on TCGA-PRAD Dataset Analysis

Addressing the clinical need for highly sensitive and reproducible needle biopsy biomarkers, we initially sought to identify optimal driver genes that exhibit both cancer-specific expression and a strong correlation with PCa aggressiveness. Through a review of the literature to identify key molecular drivers of disease progression, we selected 16 candidate genes considered to be involved in PCa prognosis prediction: ONECUT2 [15,39], EZH2 [40], BRCA2 [41], RB1 [42,43], TP53 [43,44], PTEN [43,44], FOXA1 [45], HOXB13 [46], ABCB1 [47], TUBB3 [48], MAPT [49], ENO1 [50], CKB [50], PEG10 [51], AR [52] and CCNB1 [53]. To evaluate the clinical utility of these candidates, we performed a systematic analysis using the TCGA-PRAD dataset, assessing differential expression between malignant and non-malignant tissues, correlation with pT and GG, and association with overall survival (OS) (Figure S1). First, in the diagnostic evaluation comparing malignant and non-malignant tissues, most candidates including ONECUT2 and EZH2 showed significantly different expression (*p* < 0.001), confirming their potential as cancer-specific biomarkers. Next, we assessed the correlation with disease progression and aggressiveness. EZH2 demonstrated the strongest correlation with both advanced pT stage and higher GG, followed by cell cycle-related genes such as CCNB1. ONECUT2 and BRCA2 also showed significant upregulation in locally advanced disease and high-grade tumors, indicating their association with aggressive phenotypes. Regarding prognostic value, genes reflecting cell proliferation, such as CCNB1 and TP53, showed strong statistical correlation with OS. EZH2 was also significantly associated with poor prognosis (*p* = 0.027). ONECUT2 and BRCA2 showed trends toward poor survival (*p* = 0.063 and *p* = 0.14, respectively), consistent with their role in lethal disease progression. Although CCNB1 and TP53 exhibited high statistical significance in survival analysis, we prioritized therapeutic actionability and non-redundancy of biological information for the final selection of the multiplex panel. In contrast, BRCA2 was selected because it serves as a critical theranostic biomarker for PARP inhibitors and platinum-based chemotherapy, offering direct therapeutic implications beyond mere prognostication. Similarly, EZH2 and ONECUT2 represent both key mechanistic drivers and actionable therapeutic targets for epigenetic reprogramming (e.g., EZH2 inhibitors) and lineage plasticity/neuroendocrine differentiation (e.g., small-molecule ONECUT2 inhibitors), respectively. Together, this panel offers a comprehensive “theranostic” framework, linking prognostic risk stratification directly with distinct molecular vulnerabilities.

### 4.2. ONECUT2, EZH2 and BRCA2 mRNA Expression Positively Associated with Tumor Aggressiveness

**Figure 2:**
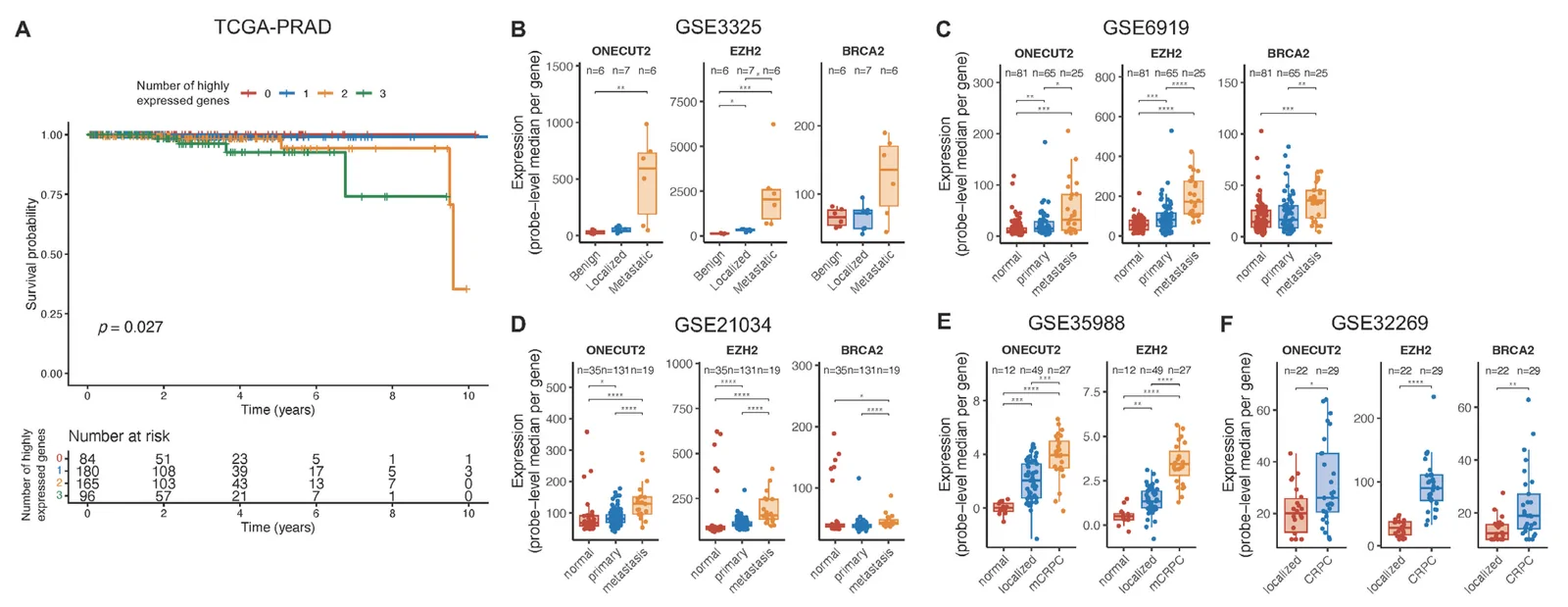
ONECUT2, EZH2, and BRCA2 mRNA expression positively correlates with tumor aggressiveness and prognosis in prostate cancer. (**A**) Kaplan–Meier overall survival curve of the TCGA-PRAD cohort, stratified by the total number of highly expressed genes (0–3). High expression (above the median) of ONECUT2, EZH2, and BRCA2 was counted as 1 each. (**B**–**D**) Relative mRNA expression of ONECUT2, EZH2, and BRCA2 in normal (benign) prostate tissue, localized prostate cancer (PCa), and metastatic PCa using data from the GSE3325 (**B**), GSE6919 (**C**), and GSE21034 (**D**) datasets. (**E**) Expression levels of ONECUT2 and EZH2 in normal prostate, localized PCa, and metastatic castration-resistant PCa (CRPC) from the GSE35988 dataset. (**F**) Expression of ONECUT2, EZH2, and BRCA2 in localized PCa and CRPC from the GSE32269 dataset. Statistical significance between groups is denoted as * *p* < 0.05, ** *p* < 0.01, *** *p* < 0.001 and **** *p* < 0.0001.

Although most of the gene expressions showed strong upregulation that was not only specific to cancer but also aggressiveness, most importantly, we found that the three-gene combination (ONECUT2, EZH2, BRCA2) significantly stratified OS (Figure 2A). To further evaluate and validate these three gene expressions across the spectrum of disease progression, we selected specific GEO datasets. GSE3325, GSE6919, and GSE21034 were chosen to encompass normal (benign), primary (localized), and metastatic PCa profiles, while GSE35988 and GSE32269 were utilized specifically for comparing primary PCa and CRPC. As shown in Figure 2B–F, ONECUT2, EZH2, and BRCA2 mRNA expression increased with not only tumor stage but also progression to metastasis. In particular, all three genes showed a marked increase in metastatic and CRPC stages, indicating a positive correlation with tumor aggressiveness.

### 4.3. Establishment of a Multiplex Real-Time RT-qPCR System for ONECUT2, EZH2, and BRCA2

**Figure 3:**
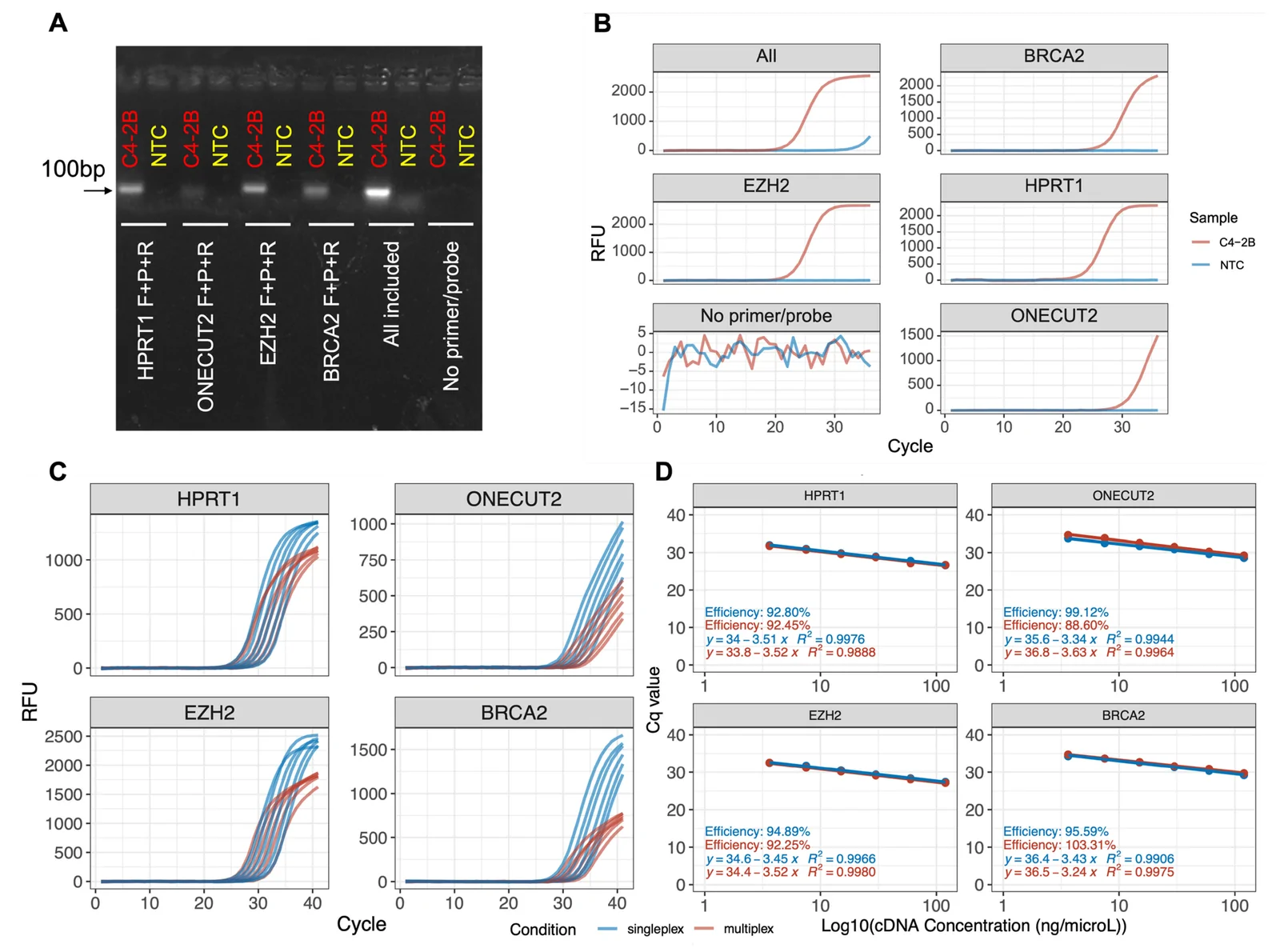
Establishment of a multiplex real-time RT-qPCR system for ONECUT2, EZH2, and BRCA2. (**A**) Agarose gel electrophoresis of RT-qPCR products. Abbreviations: F, forward primer; R, reverse primer; P, unquenched probe; NTC, no template control. The black arrow indicates the 100 bp position. (**B**) Relative fluorescence unit (RFU) amplification curves for real-time RT-qPCR using the SYBR Green method with forward primers, reverse primers, and unquenched probes for each target gene. (**C**) Comparison of RFU amplification curves between singleplex and multiplex real-time RT-qPCR assays (using primers and dual-labeled hydrolysis probes). (**D**) Standard calibration curves demonstrating comparable amplification efficiency between singleplex and multiplex real-time RT-qPCR assays.

Because of their high correlation with cancer specificity and aggressiveness for PCa or PCa patients’ survival, these genes could be a good candidate for diagnostic and/or prognostic markers for clinical implications. We established a multiplex RT-qPCR system targeting ONECUT2, EZH2 and BRCA2, as these three genes provide comprehensive, non-redundant information directly linked to precision medicine strategies. We designed primer and probe sequences for ONECUT2, EZH2, BRCA2, and the reference gene HPRT1 (Table S1). In silico analysis confirmed the absence of significant self-dimers, primer-dimers, or cross-reactivity among the oligonucleotides. We then experimentally validated the specificity of the multiplex reaction. In gel electrophoresis, although primer–dimer bands were observed in the no template control wells containing the complete primer/probe mix (in this experiment, probes did not have quenchers), no primer–dimers were produced in the presence of cDNA template (Figure 3A). Similarly, while a slight increase in RFU was noted near cycle 40 in NTC wells, no significant amplification was observed within the quantification range for all four genes either alone or in a mixture (Figure 3B). These results confirm that non-specific signals are negligible in the presence of the target template.

Importantly, the RFU amplification curves and standard curves for each gene in the multiplex assay were comparable to those obtained in singleplex assays (Figure 3C,D). These results indicate that multiplexing did not compromise assay performance, demonstrating the robustness of this real-time RT-qPCR system for targeting the expression of four genes in one reaction.

### 4.4. The Three-Gene Expression Signature Effectively Identifies Malignant Tissue and Clinically Significant Prostate Cancer

Of the 1019 needle biopsy cores collected from 270 patients, 979 samples from 255 patients were successfully analyzed by this multiplex RT-qPCR system targeting ONECUT2, EZH2 and BRCA2. The characteristics of these 255 patients are summarized in Table 1. The median follow-up time for the entire cohort was 14.0 months (IQR: 6.0–24.7). Among the 39 patients who experienced BCR, the median time to BCR was 9.0 months (IQR: 3.5–19.9).

Among the analyzed cohort, paired malignant and non-malignant samples were obtained from 116 patients (45.5%). Additionally, 100 patients (39.2%) yielded only non-malignant samples, while 30 patients (11.8%) yielded only malignant samples. Histopathological data of ex vivo prostate biopsy were unavailable for nine patients (3.5%).

**Figure 4:**
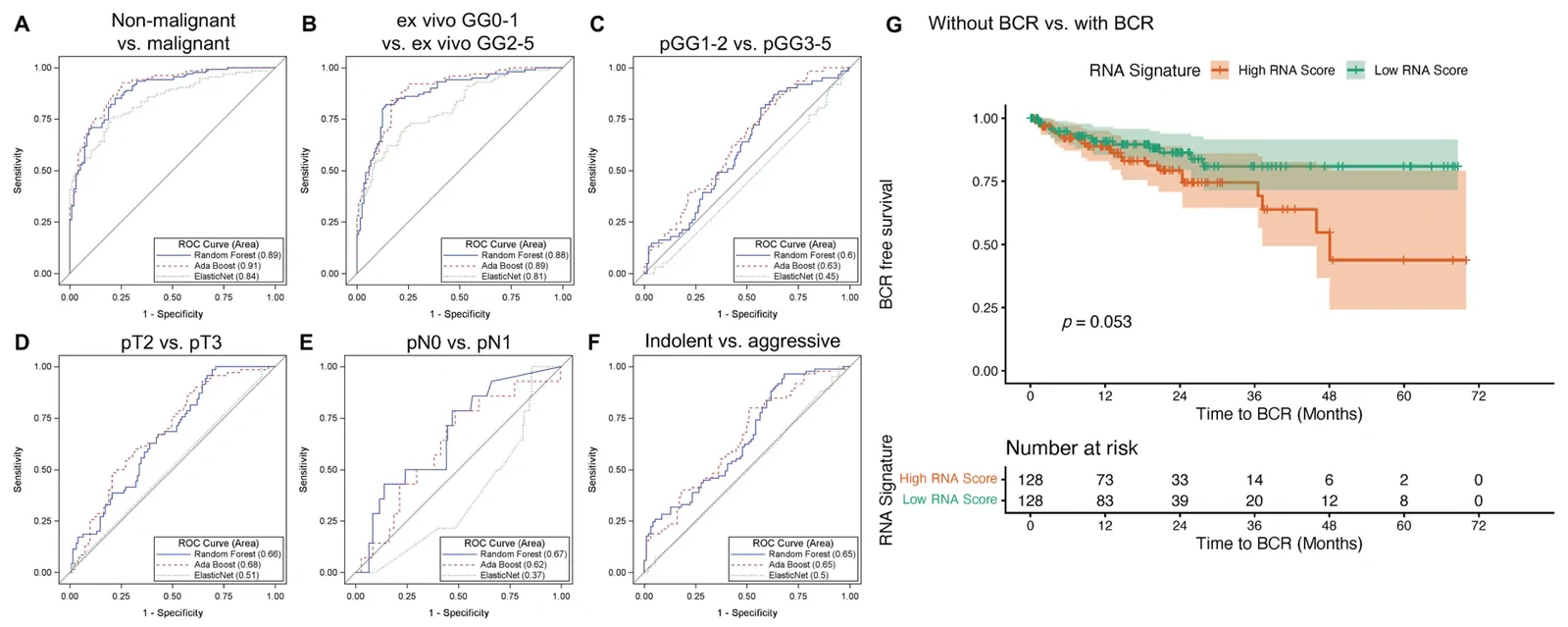
Correlation between RNA signature composed of ONECUT2, EZH2, and BRCA2 gene expression levels and pathological features in our cohort. Receiver operating characteristic (ROC) curves evaluating the diagnostic and prognostic performance of the three-gene expression signature for: (**A**) non-malignant versus malignant tissue, (**B**) ex vivo Gleason Grade Group (GG)0–1 versus ex vivo GG2–5, (**C**) pathological GG (pGG)1-2 versus pGG3–5 at the patient level, (**D**) pT2 versus pT3, (**E**) pN0 versus pN1, (**F**) indolent versus aggressive tumors, and (**G**) Kaplan–Meier BCR-free survival curves stratified by high versus low 3-gene RNA signature score. In (**A**–**F**), blue lines, red dashed lines, and green dashed lines represent the Random Forest, AdaBoost, and Elastic Net models, respectively. In (**G**), red and green lines represent the high and low RNA score groups, respectively, with shaded regions indicating 95% confidence intervals.

Next, the expression data of the three genes measured by multiplex RT-qPCR were processed using machine learning algorithms, including Random Forest, AdaBoost, and Elastic Net, to generate a predictive score, hereafter referred to as the three-gene expression signature. The signature demonstrated excellent performance in distinguishing malignant from non-malignant tissue, achieving an AUC of 0.91 (Figure 4A). Furthermore, the signature discriminated between ex vivo GG0-1 and ex vivo GG2-5 with an AUC of 0.89, highlighting its potential to identify clinically significant cancer (Figure 4B).

In contrast to its diagnostic capability, the three-gene expression signature showed limited performance in stratifying other pathological features. Specifically, AUC values for the three-gene expression signature were suboptimal for distinguishing pGG3-5 from pGG1-2 (AUC = 0.63; Figure 4C), pT3 from pT2 (AUC = 0.68; Figure 4D), pN1 from pN0 (AUC = 0.67; Figure 4E), aggressive (containing pT3, pN1 or pGG4-5) from indolent disease (AUC = 0.65; Figure 4F) and BCR-free survival, where a high RNA signature score demonstrated a modest trend toward increased recurrence risk (Log-rank *p* = 0.053, HR = 1.88, 95% CI: 0.98–3.58, C-index = 0.553; Figure 4G).

### 4.5. Potential of ONECUT2 Gene Body Methylation to Distinguish Aggressive from Indolent Features

Contrary to our initial hypothesis, the three-gene expression signature showed limited ability to distinguish aggressive pathological features from indolent ones. Given this limitation, we hypothesized that DNA methylation might offer better prognostic stratification. We utilized a previously established ONECUT2 DNA methylation dataset [15] comprising 261 samples from 80 patients to compare its diagnostic accuracy with that of this three-gene expression signature. As described in our previous study, this methylation profile targets eight CpG sites within the ONECUT2 gene body [15].

To evaluate the overall methylation status of this region, we calculated the mean methylation percentage across these eight CpG sites for each sample and used it as the input feature for our predictive models.

**Figure 5:**
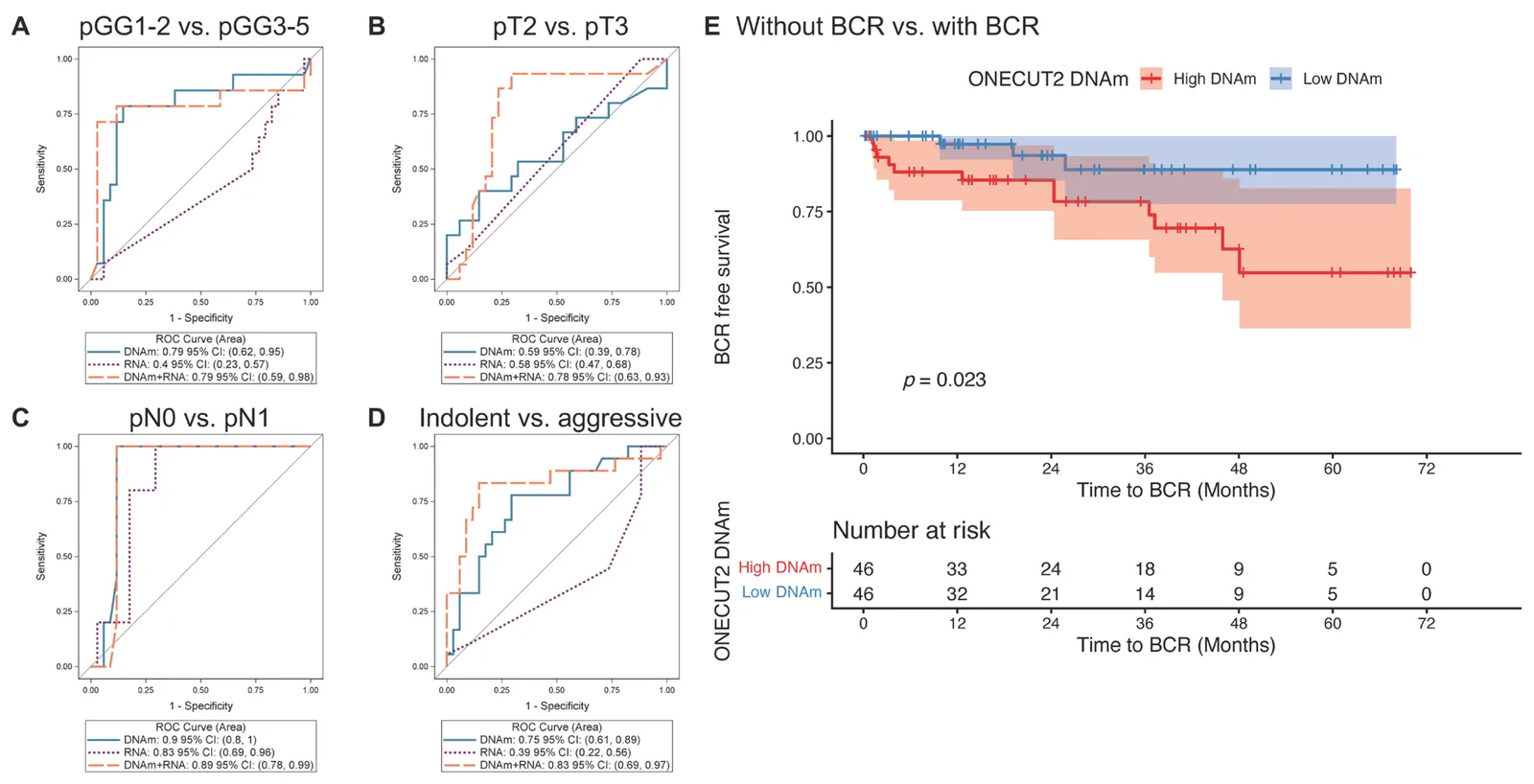
ROC curve analysis comparing the three-gene expression signature and ONECUT2 DNA methylation. Receiver operating characteristic (ROC) curves evaluating the diagnostic and prognostic performance of the three-gene expression signature and ONECUT2 DNA methylation for: (**A**) pathological GG (pGG)1–2 vs. pGG3–5, (**B**) pT2 vs. pT3, (**C**) pN0 vs. pN1, (**D**) indolent vs. aggressive disease, and (**E**) Kaplan-Meier BCR-free survival curves stratified by high versus low ONECUT2 DNA methylation. In (**A**–**D**), blue lines, purple dashed lines, and orange dashed lines represent the DNA methylation (DNAm), RNA signature (RNA), and combined (DNAm + RNA) models, respectively. In (**E**), red and blue lines represent the high and low ONECUT2 DNA methylation groups, respectively, with shaded regions indicating 95% confidence intervals.

ONECUT2 DNA methylation demonstrated superior performance in evaluating tumor aggressiveness compared to the three-gene expression signature. Specifically, it achieved significantly higher accuracy in distinguishing pGG3-5 from pGG1-2 (AUC = 0.79 vs. 0.4, *p* = 0.001), and aggressive from indolent disease (AUC = 0.75 vs. 0.39, *p* = 0.003) compared to the three-gene expression signature (Figure 5A,D and Table 2). For the identification of lymph node metastasis (pN1 vs. pN0), both modalities exhibited high predictive value, with DNA methylation and RNA achieving AUCs of 0.90 and 0.83, respectively (*p* = 0.38; Figure 5C and Table 2). In contrast, ONECUT2 gene-body DNA methylation significantly stratified BCR-free survival, with patients in the high methylation group exhibiting a significantly higher risk of recurrence compared to those in the low methylation group (Log-rank *p* = 0.023, HR = 3.90, 95% CI: 1.10–13.82, C-index = 0.654; Figure 5E and Table 2).

Furthermore, to explore the synergistic potential of these two modalities, we evaluated a combined model integrating both the three-gene expression signature and ONECUT2 DNA methylation (Table 2). Notably, while individual models for extraprostatic extension (pT3 vs. pT2) showed weak performance (AUC = 0.59 vs. 0.58), the combined model significantly improved predictive accuracy to an AUC of 0.78, representing a significant gain over the three-gene expression signature alone (ΔAUC = 0.20, *p* = 0.02; Figure 5B and Table 2). The combined model also demonstrated robust performance across other aggressive features, yielding significant improvements over the three-gene expression signature for pGG3–5 (ΔAUC = 0.39, *p* = 0.004) and aggressive disease status (ΔAUC = 0.44, *p* < 0.001).

### 4.6. Evaluation of Assay Reproducibility and Marker Stability

To assess intratumoral assay reproducibility and the robustness of our markers, we analyzed duplicate malignant samples obtained from the same patients and calculated the ICC (Figure S2). Although this analysis was limited by a relatively small number of matched pairs (*N* = 27), ONECUT2 DNA methylation demonstrated superior stability compared to the three-gene expression signature in tumor samples. Specifically, the ICC values for DNA methylation (range 0.58–0.70) were consistently higher than those for the three-gene expression signature (range 0.38–0.61). Furthermore, a paired comparison of the absolute intra-assay measurement errors (differences between replicates) revealed that the DNA methylation model had a significantly lower error rate than the RNA model when using Elastic Net (*p* = 0.034, paired Wilcoxon signed-rank test). Although this difference did not reach statistical significance in the Random Forest and AdaBoost models (*p* = 0.361 and *p* = 0.301, respectively), the overall reduction in measurement error and consistently higher ICCs suggest that DNA methylation assays provide more reproducible data for patient stratification than the three-gene expression signature, which could be caused by purity of tumor portion and heterogeneity in needle biopsies.

## 5. Discussion

Prostate needle biopsy provides essential tumor-specific details; however, biomarkers for needle biopsy remain underdeveloped, especially given the ongoing demand for highly sensitive, cost-effective, and reproducible methodologies. In this study, by integrating bioinformatic selection from TCGA-PRAD, validation across independent GEO cohorts, and experimental testing in a large ex vivo biopsy series, we aimed to establish a compact, biologically informed, and clinically actionable gene expression panel suitable for decentralized precision oncology. With this approach, we have developed and validated a simplified multiplex real-time RT-qPCR assay targeting ONECUT2, EZH2, and BRCA2 for the molecular characterization of prostate needle biopsy specimens. In parallel, we compared this expression-based signature with ONECUT2 gene body methylation, revealing distinct and complementary roles for transcriptional and epigenetic biomarkers in PCa stratification.

Our gene selection strategy prioritized biological non-redundancy and therapeutic relevance rather than purely statistical significance. Although proliferation-associated genes such as CCNB1 and TP53 demonstrated strong survival associations in TCGA-PRAD, they provide limited direct therapeutic implications [54,55]. In contrast, the final three-gene panel captures key and mechanistically distinct hallmarks of aggressive PCa, each offering direct theranostic utility: lineage plasticity and neuroendocrine differentiation with emerging small-molecular inhibitors (ONECUT2) [18], epigenetic reprogramming with actionable EZH2 inhibitors (EZH2) [56], and homologous recombination deficiency with established therapeutic vulnerability to PARP inhibitors and platinum-based chemotherapy (BRCA2) [23]. Importantly, this combination significantly stratified OS in TCGA-PRAD and showed consistent upregulation across disease progression from localized to metastatic and castration-resistant PCa in multiple GEO datasets. These findings reinforce the biological robustness of the selected markers and support their integration into a clinically oriented assay.

Technically, the multiplex RT-qPCR system demonstrated high analytical performance while requiring less sample input or RNA, which makes it well suited for needle biopsy specimens. Primer/probe design minimized cross-reactivity, and multiplex amplification showed comparable efficiency and standard curves to singleplex reactions. Non-specific amplification in no-template controls was negligible within the quantification range, indicating that multiplexing did not compromise assay specificity or sensitivity. The assay’s feasibility was further supported by the successful analysis of 979 needle biopsy cores from 255 patients, demonstrating scalability and reproducibility in a clinical-like setting. Given the practical constraints of needle biopsy tissue—limited RNA quantity and variable tumor content and heterogeneity—this robustness is critical for clinical implementation, especially since most RNA expression data in databases depend on high tumor purity achieved through microdissection [9,57].

Clinically, as our primary endpoint at the core-level, the three-gene expression signature achieved strong diagnostic performance, distinguishing malignant from non-malignant tissue with an AUC of 0.91. Moreover, it discriminated ex vivo GG2-5 from GG0-1 samples with an AUC of 0.89, indicating its ability to identify clinically significant cancer beyond purely histological assessment. Indeed, this signature shows highly competitive performance compared to traditional serum PSA (AUC = 0.72) [58], mpMRI (AUC = 0.81) [59], the 4Kscore for GS ≥ 7 PCa (AUC = 0.82) [60] and PHI (AUC = 0.708) [61]. Importantly, this core-level capability offers clear clinical utility in refining eligibility for Active Surveillance (AS) and preventing under-treatment. In routine practice, patients diagnosed with GG1 on needle biopsy are typical candidates for AS; however, due to marked tumor heterogeneity, a GG1 core may merely represent the low-grade periphery of an unsampled higher-grade tumor. Because our assay accurately discriminates GG2–5 from GG0–1 at the biopsy core level (AUC = 0.89), a histologically GG1 core exhibiting a high molecular score provides an objective, quantitative warning of underlying aggressive transcriptional programs (ONECUT2, EZH2, and BRCA2). This objective molecular readout cautions against conservative management, supporting timely confirmatory testing or definitive intervention to prevent the morphological under-grading inherent in needle biopsies.

However, despite its diagnostic strength for core-level detection, this three-gene expression signature showed limited capacity to stratify secondary patient-level pathological features, including higher pathological grade group (pGG3–5 vs. pGG1–2), extraprostatic extension (pT3), and a composite aggressive feature. These findings highlight an important distinction between cancer detection and prognostic discrimination. While mRNA expression reflects active transcriptional states and may sensitively detect tumor presence at the core-level, it may also be influenced by tumor heterogeneity, sampling variability, and microenvironmental factors [62,63,64], limiting its stability as an independent patient-level prognostic biomarker in small biopsy specimens.

In contrast, ONECUT2 gene body methylation demonstrated superior performance in stratifying pathologic grade and aggressive pathological features. Furthermore, ONECUT2 gene-body DNA methylation effectively stratified BCR-free survival (HR = 3.90, *p* = 0.023), confirming that stable DNA methylation not only reflects structural pathological aggressiveness but also serves as a robust prognostic biomarker for long-term clinical recurrence. Furthermore, methylation markers exhibited higher reproducibility, as reflected by their superior ICC compared with that of the three-gene expression signature. DNA methylation is generally more stable than RNA expression and less susceptible to rapid transcriptional fluctuations or degradation, particularly in small or partially degraded biopsy samples [65]. The observed mean ICC of 0.58–0.70 for methylation, compared with 0.38–0.61 for the three-gene expression signature, underscores its robustness for patient stratification.

These findings suggest a functional dichotomy: the three-gene expression signature is well suited for core-level cancer detection and identification of clinically significant disease to guide immediate clinical decisions (e.g., AS selection), whereas DNA methylation may better capture the stable epigenetic alteration of aggressive tumor biology.

From a translational perspective, our results support a multimodal molecular framework for biopsy evaluation. As demonstrated in our analysis, integrating DNA methylation with the RNA expression panel significantly improved the predictive accuracy (AUC) for adverse pathological features compared to the three-gene expression signature alone (Table 2). Therefore, rather than relying exclusively on a single modality, combining a compact expression panel for cancer detection with methylation-based markers for aggressiveness stratification may enhance clinical decision-making. Such a strategy aligns with precision oncology principles, enabling both biological characterization and therapeutic guidance.

Several limitations should be acknowledged. First, because this was a secondary analysis utilizing a convenience sample of all available ex vivo biopsy specimens in our institutional repository rather than a newly planned prospective cohort, the sample size was dictated by specimen availability rather than an a priori power calculation. In particular, the sample size available for head-to-head comparisons between the three-gene expression signature and DNA methylation was limited (*N* = 92), which should be considered when interpreting relative performance. To address this and prevent overfitting, we employed candidate feature pre-selection from external datasets alongside a rigorous 10-fold cross-validation framework. Second, we observed a notable rate of non-malignant cores (39.2%) despite ex vivo targeted sampling of MRI-identified lesions. This sampling limitation is largely attributable to two factors: first, our cohort composition, in which 66% of patients were clinically classified as cT1c with non-palpable or small-volume tumors; and second, the inherent technical challenges of ex vivo cognitive targeting. Although preoperative MRI identified index lesions in many cases, cognitive fusion biopsy performed on resected radical prostatectomy specimens without real-time software co-registration may lead to slight geographical mistargeting of small lesions. These factors underscore the technical challenges of biopsy sampling in localized PCa. Importantly, while this sampling error reflects the difficulty of acquiring small tumor volumes ex vivo, the assay itself demonstrated high intrinsic sensitivity (AUC = 0.91) when malignant tissue was successfully sampled, without generating false-positive signals in non-malignant cores. In routine in vivo practice, software-guided fusion biopsy would mitigate this sampling error, enhancing the assay’s clinical utility. Third, tumor heterogeneity and sampling bias inherent to needle biopsy may attenuate molecular associations. Fourth, direct external validation using public datasets (e.g., TCGA-PRAD or GEO) was technically infeasible due to fundamental platform differences (bulk RNA-sequencing/microarrays vs. our custom multiplex RT-qPCR and target-specific gene-body DNA methylation assays tailored for needle cores). Therefore, external validation in independent prospective cohorts utilizing this specific assay platform will be essential before clinical implementation.

To address these limitations and establish a truly accessible molecular grading system, our future work will focus on two main goals. First, we plan to validate our three-gene signature using in vivo needle biopsies from a broader patient population. Including a wider clinical spectrum—ranging from low-grade tumors suitable for AS to highly aggressive or metastatic disease—will help us overcome the limits of using only localized, ex vivo RP samples. Second, because DNA methylation showed better biological stability and predictive accuracy in our limited biopsy tissues, we aim to develop a multi-gene DNA methylation multiplex PCR assay. This combination of gene expression and DNA methylation markers could provide an even more reliable tool for stratifying tumor aggressiveness in clinical practice.

## 6. Conclusions

In conclusion, we established a robust, cost-effective multiplex RT-qPCR assay targeting ONECUT2, EZH2, and BRCA2 (three-gene expression signature) that performs well in cancer detection and the identification of clinically significant PCa in needle biopsy specimens. While the three-gene expression signature alone shows limited prognostic discrimination for advanced pathological features, ONECUT2 gene body methylation demonstrates superior reproducibility and aggressiveness stratification. Together, these findings support the complementary integration of transcriptional and epigenetic biomarkers into decentralized molecular grading systems for precision PCa management.

## Figures and Tables

**Table 1:** The characteristics of patients and tumors in our cohort.

Characteristic	*N* = 255
Age (years)	67 [60, 72]
Race	
Asian	25 (10%)
Black	11 (4.5%)
Others	52 (20.4%)
White	158 (64%)
Unknown	9 (3.5%)
Ethnicity	
Hispanic	32 (13%)
Non-Hispanic	211 (87%)
Unknown	12 (4.7%)
Clinical T Stage	
1c	116 (66%)
2a	34 (19%)
2b	9 (5.1%)
2c	9 (5.1%)
3a	7 (4.0%)
3b	1 (0.4%)
Preoperative PSA (ng/mL)	7.1 [5.1, 10.1]
Prostate Volume (mL)	43 [33, 62]
Biopsy Gleason Grade Group	
1	28 (11%)
2	116 (45%)
3	48 (19%)
4	31 (12%)
5	32 (13%)
NCCN Risk Group	
Very low	2 (0.8%)
Low	11 (4.4%)
Favorable intermediate	69 (28%)
Unfavorable intermediate	94 (38%)
High	70 (28%)
Very high	4 (1.6%)
Unknown	5 (2.0%)
CAPRA Score	4.00 [3.00, 6.00]
Pathological Gleason Grade Group	
1	8 (3.1%)
2	145 (57%)
3	61 (24%)
4	6 (2.4%)
5	34 (13%)
Unknown	1 (0.4%)
Pathological T Stage	
2	129 (51%)
3	96 (38%)
4	30 (12%)
Pathological N1	23 (9.1%)
Extraprostatic Extension	119 (47%)
Seminal Vesicle Invasion	31 (12%)
Intraductal Carcinoma	53 (21%)
Cribriform Architecture	132 (52%)
Bladder Neck Invasion	15 (5.9%)
Lymphovascular Invasion	24 (9.6%)
Perineural Invasion	236 (94%)

Abbreviations: CAPRA, Cancer of the Prostate Risk Assessment; NCCN, National Comprehensive Cancer Network; PSA, prostate-specific antigen; Q1, first quartile; Q3, third quartile. Notes: Data are presented as median [Q1, Q3] for continuous variables and *N* (%) for categorical variables. The denominator for percentages may vary slightly due to missing data.

**Table 2:** Predictive performance of the three-gene expression signature and DNA for pathological outcomes.

Outcome	Model	AUC/ΔAUC (95% CI)	*p* Value	Non-Events	Events
pGG1-2 vs. pGG3-5	DNAm	0.79 (0.62, 0.95)		34	14
	RNA	0.40 (0.23, 0.57)		34	14
	Combined	0.79 (0.59, 0.98)		34	14
	ΔAUC (DNAm-RNA)	0.38 (0.15, 0.62)	*p* = 0.001	34	14
	ΔAUC (Combined-RNA)	0.39 (0.12, 0.65)	*p* = 0.004	34	14
pT2 vs. pT3	DNAm	0.59 (0.39, 0.78)		34	15
	RNA	0.58 (0.47, 0.68)		34	15
	Combined	0.78 (0.63, 0.93)		34	15
	ΔAUC (DNAm-RNA)	0.01 (−0.19, 0.21)	*p* = 0.92	34	15
	ΔAUC (Combined-RNA)	0.20 (0.03, 0.37)	*p* = 0.02	34	15
pN0 vs. pN1	DNAm	0.90 (0.8, 1)		34	5
	RNA	0.83 (0.69, 0.96)		34	5
	Combined	0.89 (0.78, 0.99)		34	5
	ΔAUC (DNAm-RNA)	0.07 (−0.08, 0.22)	*p* = 0.38	34	5
	ΔAUC (Combined-RNA)	0.06 (−0.08, 0.19)	*p* = 0.4	34	5
Indolent vs. aggressive	DNAm	0.75 (0.61, 0.89)		34	18
	RNA	0.39 (0.22, 0.56)		34	18
	Combined	0.83 (0.69, 0.97)		34	18
	ΔAUC (DNAm-RNA)	0.36 (0.12, 0.6)	*p* = 0.003	34	18
	ΔAUC (Combined-RNA)	0.44 (0.22, 0.66)	*p* < 0.001	34	18

Abbreviations: AUC, area under the receiver operating characteristic curve; ΔAUC, difference in AUC; CI, confidence interval; DNAm, DNA methylation; pGG, pathological Gleason Grade Group; pN, pathological regional lymph node stage; pT, pathological primary tumor stage. Notes: The ‘DNAm’ model is based on ONECUT2 DNA methylation. The ‘RNA’ model is based on the three-gene expression signature (ONECUT2, EZH2, and BRCA2). ‘Combined’ represents the integrated model combining both DNAm and RNA modalities. *p*-values indicate the statistical significance of the difference in predictive performance between the indicated models. Predictive performance was evaluated using three distinct machine-learning algorithms (AdaBoost, Elastic Net, and Random Forest). Values in parentheses represent 95% confidence intervals.

## Data Availability

The datasets analyzed during the current study, including TCGA-PRAD and GEO cohorts, are available in public-domain resources (The Cancer Genome Atlas and Gene Expression Omnibus under accession numbers GSE3325, GSE6919, GSE21034, GSE35988, and GSE32269). The clinical and newly generated experimental datasets (multiplex RT-qPCR and DNA methylation data) presented in this study are available on request from the corresponding author due to patient privacy and ethical restrictions.

## Supplementary Materials

The following supporting information can be downloaded at: https://www.mdpi.com/article/10.3390/cancers18182962/s1.

## Abbreviations

AS	Active Surveillance
AUC	Area under the receiver operating characteristic curve
BCR	Biochemical recurrence
CAPRA	Cancer of the Prostate Risk Assessment
CRPC	Castration-resistant prostate cancer
GEO	Gene Expression Omnibus
GG	Gleason Grade Group
ICC	Intraclass correlation coefficient
IQR	Interquartile range
NCCN	National Comprehensive Cancer Network
OS	Overall survival
PARP	Poly (ADP-ribose) polymerase
PCa	Prostate cancer
pN	pathological N
PSA	Prostate-specific antigen
pT	pathological T
RFU	Relative fluorescence units
TCGA-PRAD	The Cancer Genome Atlas Prostate Adenocarcinoma
TPM	Transcripts Per Million
