# Supplementary material for: A Multiplex RT-QPCR Assay of ONECUT2, EZH2, and BRCA2 for the Detection of Clinically Significant Prostate Cancer in Needle Biopsies
Source: Cancers (Basel). 2026 Sep 14;18(18):2962. doi: 10.3390/cancers18182962 (PMC13604541; doi:10.3390/cancers18182962)
Supplement: Supplementary file 1 [file cancers-18-02962-s001.zip › Supplementary information.pdf]

Additional file 1

Figure S1.

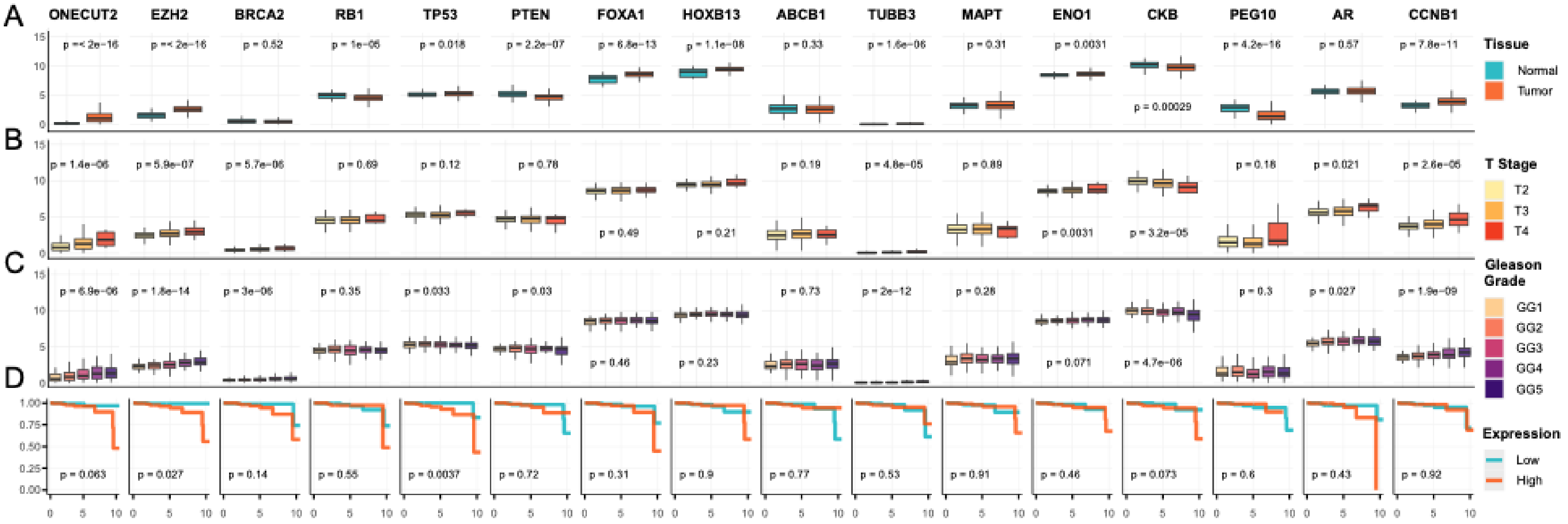

Comprehensive in silico analysis of 16 candidate genes in the TCGA-PRAD cohort. (A) Relative mRNA expression levels of the 16 candidate genes compared between non-malignant (Normal) and malignant (Tumor) prostate tissues (Wilcoxon rank-sum test). (B, C) Association of the relative mRNA expression levels with advanced clinicopathological features, including pathological T stage (B) and Gleason score (C) (Kruskal-Wallis H test). (D) Kaplan-Meier analysis of overall survival probability for prostate cancer patients, stratified by the median relative expression level of each gene (High vs. Low expression). P-values were determined using the log-rank test.

Figure S2.

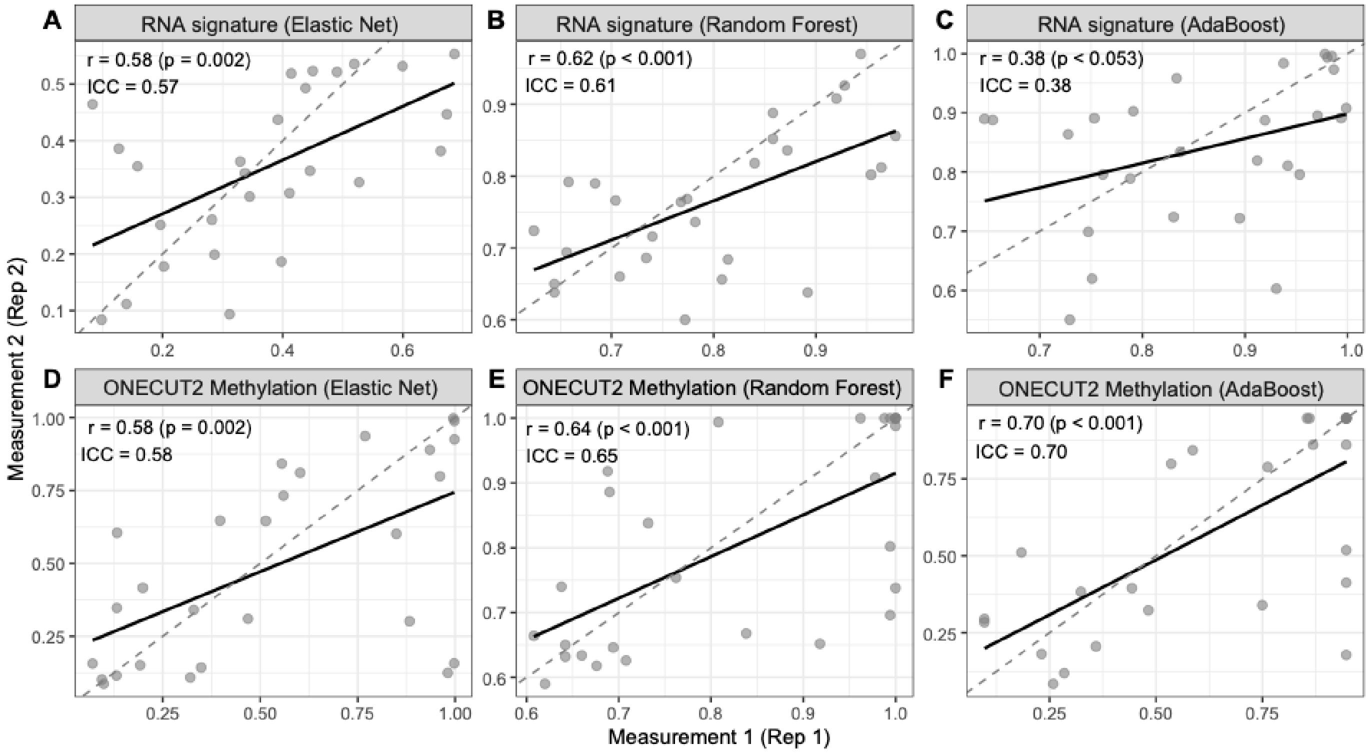

Intraclass correlation coefficient (ICC) evaluating assay reproducibility for the RNA signature (A-C) and ONECUT2 DNA methylation (D-F)

Table S1: Primers and probes sequences

HPRT1	Forward	TGGACTAATTATGGACAGGACTGA
	Reverse	TGTGATGAAGGAGATGGGAGGC
	Probe	AGCACACAGAGGGCTACAAT
ONECUT2	Forward	GATGTGGAAGTGGCTTCAGGA
	Reverse	TTCCAGCGCATGTCCGCCTTA
	Probe	TTTGCGTTTGCACGCTGCC
EZH2	Forward	CAGCTGAAAAAGGACGGCTC
	Reverse	CAACCCTGTGATCATCCACGGC
	Probe	ACAAGGGCACGAACTGTCAC
BRCA2	Forward	TGTACACATGTAACACCACAAAGAG
	Reverse	GTATGTGGGAGTTTGTTTCATACA
	Probe	TTGGTGTCTGACGACCCTTC
